# Completion of Volume Third

**Published:** 1864-07

**Authors:** 


### EDITORIAL DEPARTMENT.

### COMPLETION OF VOLUME THIRD.

This number completes our third volume, and seals up the record, so far as opportunity to change or improve our past is concerned; three years with our Journal "have now become history." It will be remembered that our commencement was in those perilous days of commencing rebellion or revolution, when one thought filled all minds, and one effort engaged all hands; when news of the war overshadowed all other intelligence, and left scarce a niche to be occupied by any other adventurer. At this time everything stood still, or moved slowly backwards in expectation of some great change. It was a great thing to be born at such a time; to come up to life under such circumstances of peril and danger. It was a great thing to live, after we were born, and stand a trembling witness of the death of our superiors; yes, it was a great thing, and speaks volumes for us, which perhaps it might not be in good taste to write up. Our past is fully before our attentive readers, though it is to be presumed we have a private history not altogether familiar to them—not to be fully understood until the "secrets of all hearts are made known." However, with many changes between hope and despondency we have made thus far our monthly returns, for which we have been more indebted to "foreign trade," than to to any "internal revenue."

At present under any ordinary circumstances, we should be an established "institution," but what can now be established which depends in any degree upon the price of gold? We have (as an institution) mourned over the fact that our medical gospel could not be offered the profession "freely, without money and without price;" but no amount of tears can avail "to wash away the stains" of debt, or obliterate the printer's bills. Gold or its equivalent in "green-backs," is the only available remedy. This is history, and not written to delinquent subscribers, since we have but few upon our list, and these we expect to hear from, before our next issue.

We are as yet unable to announce the price of volume four, for it is impossible to ascertain upon what terms it can be published. We desire to receive only sufficient to pay the publication, and we hardly as yet know which is best, to raise the price to meet the immense increase in cost or to raise on the price of professional services and let that of the Journal remain unchanged. Meantime, while we are undecided is a good opportunity to subscribe, or pay up old dues. A few physicians have received our Journal from the beginning, and have as yet never made any suitable acknowledgments; but under numerous embarrassments, we are not disposed to "look back" "after having put our hands to the plow."

We desire to express our most hearty thanks to our contributors and correspondents for their kind and generous co-operation, and trust they will continue to furnish their welcome articles, thus greatly assisting to make the Journal of practical value to the general practitioner, and more fully constituting it a medium of professional communication. We also acknowledge our obligations to the profession generally for their generous support and kind indulgence, for their numerous expressions of confidence and favor, and shall be but too happy, if we are able to merit their continued approbation.

With past experience as a guide, and with constant, untiring effort, we shall offer our future Journal, with the hope that it may grow better, as it grows older.

